# Effectors of Plant Necrotrophic Fungi

**DOI:** 10.3389/fpls.2021.687713

**Published:** 2021-06-04

**Authors:** Dandan Shao, Damon L. Smith, Mehdi Kabbage, Mitchell G. Roth

**Affiliations:** Department of Plant Pathology, University of Wisconsin – Madison, Madison, WI, United States

**Keywords:** necrotrophic fungi, effector, programmed cell death, hypersensitive response, necrosis-inducing activity, defense suppression, sRNA effectors

## Abstract

Plant diseases caused by necrotrophic fungal pathogens result in large economic losses in field crop production worldwide. Effectors are important players of plant-pathogen interaction and deployed by pathogens to facilitate plant colonization and nutrient acquisition. Compared to biotrophic and hemibiotrophic fungal pathogens, effector biology is poorly understood for necrotrophic fungal pathogens. Recent bioinformatics advances have accelerated the prediction and discovery of effectors from necrotrophic fungi, and their functional context is currently being clarified. In this review we examine effectors utilized by necrotrophic fungi and hemibiotrophic fungi in the latter stages of disease development, including plant cell death manipulation. We define “effectors” as secreted proteins and other molecules that affect plant physiology in ways that contribute to disease establishment and progression. Studying and understanding the mechanisms of necrotrophic effectors is critical for identifying avenues of genetic intervention that could lead to improved resistance to these pathogens in plants.

## Introduction

Plant pathogens are often categorized based on their relationship with host plants. These categories include biotrophs, which infect living plants with the objective of suppressing the plant immune system and acquiring nutrients from living cells; necrotrophs, which infect living plants with the objective of killing the plant upon or shortly after infection and acquiring nutrients from dead or dying tissues; and hemibiotrophs, which infect living plant tissues akin to biotrophs to first establish infection before “switching” to necrotrophy and killing the plant (Giraldo and Valent, 2013). Despite these seemingly distinct definitions, arguments can be made that trophic designations of some of these pathogens are convoluted as we learn more about the complexities of their interactions with a given host plant (Kabbage et al., 2013, 2015; Toruño et al., 2016). A common theme that unifies plant-associated fungal pathogens despite their different colonization and nutrient acquisition strategies, is the use of effectors during plant colonization (Giraldo and Valent, 2013). While more frequently studied in biotrophic and hemibiotrophic fungi, effectors in necrotrophic fungi have only recently begun to receive significant attention. This review discusses the roles of effectors from necrotrophic and late-stage hemibiotrophic fungi in disease development, including induction of plant cell death, suppression of plant immunity, and the activation of susceptibility genes (inverse gene-for-gene relationship).

As efficient plant killers, necrotrophic fungi are infamous for causing significant losses in the field and in storage worldwide, and diseases that cause annual threats to food security (Fones et al., 2020). Managing diseases caused by necrotrophic fungi requires multiple approaches, though fungicide applications are the most common approach currently used. The heavy reliance on spray regimes is costly, poses environmental challenges, and can lead to the emergence of resistant populations (Brent and Hollomon, 2007). Therefore, control strategies through improving genetic resistance are desirable, yet our understanding of the molecular intricacies between the plant and necrotrophs lags behind their biotrophic counterparts. Notably, a broad understanding of the role of effectors in the pathogenic development of these fungi is lacking. As their roles in this pathogenic system are clarified, necrotrophic effectors and their plant targets will become targets for manipulation to confer resistance to these pathogens.

Effector proteins are narrowly defined as small, cysteine rich, secreted proteins used by pathogens to manipulate plant cellular responses to the benefit of the pathogen. Effectors from plant biotrophic/hemibiotrophic fungi have been studied by generations of plant pathologists since the gene-for-gene theory was put forward by Flor (1971), and their ability to suppress plant immunity, manipulate plant physiology, and be recognized by host defense mechanisms is well documented (Koeck et al., 2011). In contrast, necrotrophs were previously thought to use a brute-force approach, including the deployment of large repertoires of cell wall degrading enzymes (CWDE) and broad-spectrum toxins to kill plant cells in advance of fungal growth, obtaining nutrients from dead tissue. However, recent advances in genome and transcriptome sequencing, and computational prediction tools suggest there are hundreds of putative effectors present in necrotrophic genomes (Sperschneider et al., 2016; Derbyshire et al., 2017; Lopez et al., 2018; Le Marquer et al., 2019). The surprisingly large number of effectors identified in necrotrophs indicates that the mechanisms deployed by necrotrophic fungi are more intricate than initially hypothesized. Indeed, functional studies of effectors referenced in this review support this notion, and suggest a clear contribution of these molecules to the pathogenic success of fungal necrotrophs.

Herein, we broadly discuss the participation of effectors from necrotrophic and late stage hemibiotrophic fungi in disease development, plant cell death modulation, and both the suppression and the hijacking of plant immune responses. In addition to proteinaceous effectors, we also discuss the recent discovery of small RNAs produced by necrotrophic fungi and their role in disease establishment.

## Plant Cell Death-Inducing Effectors

By definition, necrotrophs kill host cells and acquire nutrients from dead cells. It is thus reasonable to speculate that necrotrophic fungi may utilize effectors to coopt host programmed cell death (PCD). Indeed, many examples of effectors from necrotrophic fungi have been reported for their cell death-inducing activities. Inducing cell death in plants can release many plant-derived compounds that are toxic to fungi, so it is also important to consider fungal proteins involved in tolerating this environment. These detoxification mechanisms were the subject of another recent review on necrotrophic fungi (Westrick et al., 2021), and will not be covered here.

### Necrotrophic Effectors That Hijack R-Mediated Resistance

The hypersensitive response (HR) is a localized cell death program triggered by the recognition of effectors by plant resistance (R) proteins to confer resistance against biotrophs and hemibiotrophs, and is commonly referred to as Effector-Triggered Immunity (ETI) (Jones and Dangl, 2006). Remarkably, this genetic program can be hijacked by necrotrophs to their own benefit, considering their trophic lifestyle. This phenomenon is termed effector-triggered susceptibility (ETS) due to the activation of plant defense responses often including cell death, leading to susceptibility to necrotrophs (Table 1; Williams and Dickman, 2008). These necrotrophic effectors were initially termed “host selective toxins” (HST), and are typically effective in a narrow range of plant hosts (Tan et al., 2010).

**TABLE 1:** Effectors that induce plant cell death in an inverse gene-for-gene manner.

Pathogen name	Effector	Protein domain/function	Plant susceptibility gene or locus	References
*Pyrenophora tritici-repentis*	PtrToxA	arginine-glycine-aspartic acid (RGD) motif	Tsn1	Manning and Ciuffetti, 2005, 2015; Manning et al., 2007; Faris et al., 2010
	PtrToxB	N/A	Tsc2	Friesen and Faris, 2004; Figueroa et al., 2015
*Parastagonospora nodorum*	SnToxA	arginine-glycine-aspartic acid (RGD) motif	Tsn1	Friesen et al., 2006
	SnTox1	N/A	Snn1	Liu et al., 2004, 2012, 2016
	SnTox2	N/A	Snn2	Friesen et al., 2007
	SnTox3	N/A	Snn3	Liu et al., 2009
	SnTox4	N/A	Snn4	Abeysekara et al., 2009
	SnTox5	N/A	Snn5	Friesen et al., 2012
	SnTox6	N/A	Snn6	Gao et al., 2015
	SnTox7	N/A	Snn7	Shi et al., 2015
	SnTox8	N/A	Snn8	Faris et al., 2007
*Cochliobolus heterostrophus*	ChToxA	N/A	unknown	Lu et al., 2015
*Cochliobolus sativus*; (*Bipolaris sorokiniana*)	BsToxA	N/A	Tsn1	McDonald et al., 2018
*Corynespora cassiicola*	cassiicolin	N/A	unknown	de Lamotte et al., 2007
*Cochliobolus victoriae*	victorin	N/A	Vb in oat, LOV1 in *Arabidopsis*	Wolpert et al., 1985; Lorang et al., 2007

An excellent example of this is found in the tan spot pathogen of wheat, *Pyrenophora tritici-repentis*. *P. tritici-repentis* secretes two effectors, PtrToxA (Tuori et al., 1995) and PtrToxB (Friesen and Faris, 2004), that are critical for disease development. Susceptibility in wheat is conferred by the Tsn1 gene, a classical plant R gene with conserved features such as a serine/threonine protein kinase (S/TPK) domain, nucleotide binding site (NBS), and leucine rich repeat (LRR) domain. Tsn1 is required for plant susceptibility to PtrToxA, although Tsn1 does not directly interact with PtrToxA (Faris et al., 2010). PtrToxA translocates into plant cells possibly through the recognition of arginine-glycine-aspartic acid (RGD) motif in the protein by a plant surface receptor (Manning and Ciuffetti, 2005) and eventually localizes to chloroplasts leading to light-dependent reactive oxygen species (ROS) accumulation and cell death (Ciuffetti et al., 2010). To date, the precise role of Tsn1 in ToxA sensitivity is unknown, though the evidence is clear that Tsn1 plays a strong role in sensitivity to PtrToxA. PtrToxA sensitivity in plants lacking Tsn1 have also been reported (Manning and Ciuffetti, 2015), suggesting the presence of other susceptibility factors linked to PtrToxA. Similarly, PtrToxB also requires wheat genotypes which possess the single dominant susceptibility locus Tsc2 (Friesen and Faris, 2004), though the specific PtrToxB sensitivity gene in this locus remains elusive (Corsi et al., 2020). In contrast to PtrToxA, PtrToxB appears to function extracellularly in the plant apoplast (Figueroa et al., 2015). However, the extracellular interactors and specific mode of action of PtrToxB are yet to be identified.

*Parastagonospora nodorum* is another necrotroph with host-selective effectors that target specific host genotypes of wheat and other cereals, causing *P. nodorum* leaf blotch (Solomon et al., 2006). Nine host-specific effectors from *P. nodorum* and their corresponding host susceptibility loci have been identified and characterized (reviewed by Cowger et al., 2020). SnTox1 was the first HST identified in *P. nodorum* that requires a single host susceptibility locus Snn1 to confer susceptibility (Liu et al., 2004). Snn1 genes allow recognition of SnTox1 in a light-dependent manner, triggering hallmarks of PCD including an oxidative burst, strong up-regulation of pathogenesis-related (PR) genes, and DNA laddering (Liu et al., 2012). Notably, SnTox1 was also shown to have a chitin binding domain, which is thought to play a role in protecting fungal cell walls from degradation by plant chitinases (Liu et al., 2016). This feature may also be important to prevent the release of chitin monomers, which are recognized by pattern recognition receptors (PRRs) and subsequent activation of immune responses (discussed in section 2: Plant Immunity-suppressing Effectors). Another *P. nodorum* effector, SnToxA, was shown to be almost identical to PtrToxA from *P. tritici-repentis* and shares the same host susceptibility gene, Tsn1 (Friesen et al., 2006), suggesting that *P. tritici-repentis* acquired ToxA from *P. nodorum* by horizontal gene transfer (Friesen et al., 2006). Seven additional *P. nodorum* HSTs, SnTox2, SnTox3, SnTox4, SnTox5, SnTox6, SnTox7, and SnTox8, have been identified along with their corresponding susceptibility loci, Snn2 (Friesen et al., 2007), Snn3 (Liu et al., 2009), Snn4 (Abeysekara et al., 2009), Snn5 (Friesen et al., 2012), Snn6 (Gao et al., 2015), Snn7 (Shi et al., 2015), and Snn8 (Faris et al., 2007), respectively. Due to the complex nature of the wheat genome, specific genes within susceptibility loci that are responsible for the susceptibility to *P. nodorum* effectors remain unknown. However, these studies report that the SnTox2-Snn2, SnTox4-Snn4, SnTox5-Snn5, and SnTox6-Snn6 interactions are all dependent on light and very likely share the similar downstream pathways leading to the induction of PCD, thus subverting this plant resistance mechanism for the benefit of the necrotrophic pathogen.

In the maize pathogen *Cochliobolus heterostrophus*, a ToxA-like gene (ChTOXA) was found to share both sequence and structure similarities with PtrToxA, with the exception of the putative translocation RGD motif (Lu et al., 2015). It induces light-dependent cell death in sensitive maize lines, however, the maize gene responsible for susceptibility to ChToxA is yet to be identified (Lu et al., 2015). Recent genome analyses of three *C. sativus* isolates (anamorph *Bipolaris sorokiniana*), the causal agent for multiple diseases in wheat and barley, have also revealed the presence of a ToxA-like gene that shares homology with PtrToxA and SnToxA (McDonald et al., 2018). Pathogenicity assays show that isolates harboring ToxA genes are more virulent on Tsn1 wheat genotypes (McDonald et al., 2018), suggesting ToxA from *C. sativus* functions similarly to other ToxA effectors.

*Corynespora cassiicola* is a necrotroph that produces a proteinaceous HST known as cassiicolin, a small, secreted, cysteine-rich phytotoxic protein (de Lamotte et al., 2007). *C. cassiicola* is the causal agent of leaf spot or leaf fall disease on many economically important crops, including rubber tree, cocoa, and soybean (Silva et al., 2000). At least seven Cas genes encoding isoforms of cassiicolin have been identified in various *C. cassiicola* isolates (Cas1 to Cas7) (Déon et al., 2014; Lopez et al., 2018). In a recent study, a *cas1* mutant generated from a highly aggressive isolate lost all virulence in susceptible rubber tree clones (Ribeiro et al., 2019). Quantitative trait loci (QTL) analysis has identified potential loci associated with the rubber tree susceptibility to cassiicolin and other *C. cassiicola* effectors (Ribeiro et al., 2019), however, specific sensitivity genes within these QTL are yet to be explored.

One example of a non-traditional effector is victorin, produced by *Cochliobolus victoriae*, the causal agent of Victoria blight in oats. Victorin functions within a narrow range of plant hosts, so it is considered a HST. Victorin is non-traditional because it is a mixture of hexapeptides that were previously thought to be synthesized independent of ribosomes. Surprisingly, recent evidence revealed that victorin is in fact ribosomally synthesized (Kessler et al., 2020). The Vb gene in oat (Wolpert et al., 1985) and LOV1 gene in *Arabidopsis thaliana* determines the sensitivity to victorin (Lorang et al., 2007). Similar to susceptibility genes against *S. nodorum* and *P. tritici-repentis*, LOV1 in Arabidopsis also encodes a characteristic R gene with NBS-LRR domains (Lorang et al., 2007) and the recognition of victorin elicits apoptotic-like cell death which facilitates the infection of *C. victoriae* as a necrotroph. The Pc-2 gene in oat confers disease resistance against rust pathogen *Puccinia coronata*, and is tightly linked to the Vb gene, complicating breeding efforts against both rust and blight diseases (Meehan and Murphy, 1946; Litzenberger, 1949). Pc-2 and Vb genes are now considered to be the same gene that confers resistance to *Puccinia coronata*, but susceptibility to C. *victoriae* (Wolpert et al., 2002; Lorang et al., 2012). This constitutes a remarkable example of how necrotrophs can evolve mechanisms to highjack R genes against biotrophs to their own benefit. Additional non-traditional HSTs are discussed in section 1.6.

### Effectors That Hijack PCD Pathways

Besides inducing PCD via an inverse gene-for-gene manner, necrotrophic effectors can also target or hijack specific components of the PCD pathway. Upon pathogen attack, plants often increase the level of cytosolic calcium, which stimulates calcium-dependent signaling mechanisms that mediate plant defense responses, often culminating in HR or PCD (Lam et al., 2001). Thus, mimicking such responses can be beneficial to necrotrophs.

Endopolygalacturonases are commonly secreted by fungi as CWDE, releasing oligogalacturonides as by-products. However, one endopolygalacturonase purified from *Sclerotinia sclerotiorum* also increases intracellular calcium and triggers hallmarks of apoptosis-type cell death, including cytochrome c release from mitochondria and activation of caspase 9-like and caspase 3-like enzymes in soybean cells (Zuppini et al., 2005). In contrast, the oligogalacturonides byproducts were unable to initiate PCD (Binet et al., 2001), revealing that the *S. scleortiorum* endopolygalacturonase alone is the trigger of PCD, independent of its presumed enzymatic activity (Zuppini et al., 2005).

Sspg1d is another endopolygalacturonase from *S. sclerotiorum* that contributes to PCD induction in plants (Wang et al., 2009). Instead of functioning as a CWDE, Sspg1d specifically interacts with the C2 domain of the canola IPG-1 protein. C2 domain proteins have calcium-binding affinity and are likely involved in calcium-dependent signal transduction (Evans et al., 2004) and defense responses (Laxalt et al., 2001). IPG-1 subcellular localization is determined by calcium concentrations, indicating a role in calcium dependent signaling. IPG-1 is also highly upregulated during a compatible interaction with *S. sclerotiorum*, leading to susceptibility. As several C2 domain proteins were reported to be PCD regulators (Yang et al., 2007) and another known endopolygalacturonase of *S. sclerotiorum* induced PCD in host plant (Zuppini et al., 2005), Sspg1d is proposed to promote PCD by interfering with the activity of IPG-1 as a negative regulator of PCD (Wang et al., 2009). However, not all endopolygalacturonases have the capacity to coopt plant cell death components, and likely function as classical CWDEs. For instance, endopolygalacturonases from *Botrytis cinerea* (BcPG1 and BcPG2) induce necrotic cell death by provoking a loss of cell wall integrity, as is the case of many CWDEs produced by fungal pathogens (Kars et al., 2005).

### Cell Death Inducing Effectors Containing Conserved Domains

The term “necrosis-inducing” can be misleading, as necrosis typically refers to cell death involving non-physiological or non-programmed events due to overwhelming injury. Effectors that contain the necrosis and ethylene-inducing peptide (NEP) domain have been shown to induce cell death in plants, and despite necrosis in the name, this mechanism appears to be physiological for some NEPs (Table 2).

**TABLE 2:** Effectors with necrosis-inducing or other known domains.

Pathogen name	Effector	Protein domain/function	Plant target	References
*Sclerotinia sclerotiorum*	SsNep1	necrosis and ethylene-inducing peptides (NEP)	unknown	Dallal-Bashi et al., 2010
	SsNep2	NEP	unknown	Dallal-Bashi et al., 2010
	SsCP1	Cerato-platanin protein	unknown	Yang G. et al., 2018
*Botrytis cinerea*	BcSp1	Cerato-platanin protein	unknown	Frías et al., 2011
	BcGs1	glucan 1,4-alpha-glucosidase	unknown	Zhang et al., 2015
	BcXyn11A	xylanase	unknown	Noda et al., 2010; Frías et al., 2019
	BcXyl1	xylanase	unknown	Yang Y. et al., 2018
	BcXYG1	xyloglucanase	unknown	Zhu et al., 2017
	BcCrh1	Congo red hypersensitivity transglycosylase	unknown	Bi et al., 2020
	BcIEB1	IgE binding protein	osmotin	Frías et al., 2016; González et al., 2017
*Botrytis elliptica*	BeNEP1	NEP	unknown	Staats et al., 2007
	BeNEP2	NEP	unknown	Staats et al., 2007
*Colletotrichum higginsianum*	ChNLP1	NEP	unknown	Kleemann et al., 2012
	ChNLP2	NEP	unknown	Kleemann et al., 2012
*Heterobasidion annosum*	HaCPL2	Cerato-platanin protein	unknown	Chen et al., 2015
*Rhizoctonia solani*	AGLIP1	lipase domain	unknown	Li et al., 2019

The first NEP (Nep1) was isolated from *Fusarium oxysporum* infecting a coca plant (*Erythroxylum coca*) (Bailey, 1995), and Nep1 also triggers cell death in *Arabidopsis* (Bae et al., 2006). Two *F. oxysporum* Nep1 homologs were identified in *S. sclerotiorum* and referred to as SsNep1 and SsNep2 (Dallal-Bashi et al., 2010). SsNep2 was highly expressed during infection on *Brassica napus* leaves and caused PCD that is dependent on calcium signaling (Dallal-Bashi et al., 2010). Nep-like proteins (NLPs) appear to be conserved in many filamentous fungi, some of which have cell death-inducing qualities and others do not (Oome and Van den Ackerveken, 2014). Many NLPs have a calcium binding pocket and may also be influenced by intracellular calcium concentrations (Oome and Van den Ackerveken, 2014), suggesting that these molecules may influence plant cells into committing suicide. In the lily pathogen *Botrytis elliptica*, two NLPs (BeNEP1 and BeNEP2) were identified and display cell death-inducing activity in a range of dicots, but not monocots, including its host lily (Staats et al., 2007). Therefore, NLPs are not essential virulence factors or HSTs for *B. elliptica* on lily, but may be a horizontally acquired feature that led to a competitive advantage allowing colonization of additional hosts (Staats et al., 2007). In hemibiotrophic fungus *Colletotrichum higginsianum*, six NLPs were identified (ChNLPs) based on the presence of NEP domains. Among them, only ChNLP1 and ChNLP2 were expressed exclusively during the transition to necrotrophy and induced potent cell death when overexpressed in *Nicotiana benthamiana* (Kleemann et al., 2012). Additional NLPs from *Colletotrichum orbiculare* and *Fusarium virguliforme* are also reported to induce cell death in tobacco and soybean, respectively (Yoshino et al., 2012; Chang et al., 2016). The postharvest pathogen *Penicillium expansum* contains two NLP effectors, PeNLP1 and PeNLP2 that cause cell death when transiently expressed in *N. benthamiana* (Levin et al., 2019). PeNLP1 is highly induced compared to PeNLP2 during infection of apple fruit, and deletion of PeNLP1, but not PeNLP2, led to a significant reduction in lesion size (Levin et al., 2019).

Cerato-platanin family proteins (CPPs) are another secreted protein family found only in filamentous fungi and reported to cause phytotoxicity, including in *B. cinerea* (Frías et al., 2011), *S. sclerotiorum* (Yang G. et al., 2018), *Heterobasidion annosum* (Chen et al., 2015), and *Magnaporthe oryzae* (Yang et al., 2009). BcSpl1, a CPP from *B. cinerea*, is the sixth most abundant protein in the secretome of this fungus (Frías et al., 2011), and mutants lacking BcSpl1 show reduced virulence compared to wild-type strains. The infiltration of purified BcSpl1 protein causes rapid cell death in tomato, tobacco, and *Arabidopsis* leaves in a dose-dependent manner, and this cell death response was accompanied by hallmarks of the HR response; autofluorescence, ROS burst, electrolyte leakage, and cytoplasm shrinkage (Frías et al., 2011). Furthermore, the BcSpl1 activity requires the membrane-bound co-receptor BAK1 for full activity, which is known to enhance immune signaling responses in plants (Schwessinger and Ronald, 2012). The detection of cell surface receptors associated with PCD suggest a physiological and subtle cell death induction by necrotrophs and their effectors. This supports a growing number of hypotheses that some necrotrophic fungi have evolved mechanisms to hijack and induce plant PCD for their own benefit of colonizing and feeding off the dead plant tissues (Kabbage et al., 2013).

Another CPP was recently identified in *S. sclerotiorum* (SsCP1), a close relative of *B. cinerea*, as an important contributor to virulence (Yang G. et al., 2018). Similar to BcSpl1, SsCP1 induces cell death when transiently overexpressed in *N. benthamiana* in a dose-dependent manner (Yang G. et al., 2018). Interestingly, SsCP1 also interacts with PR1 in the apoplast, presumably inhibiting the antifungal activity of PR1 and promoting infection (Yang G. et al., 2018). A CPP-like protein HaCPL2 was also found in the basidiomycete *Heterobasidion annosum sensu stricto*, which is a necrotrophic fungal pathogen of Scot pine (*Pinus sylvestris*) (Chen et al., 2015). HaCPL2 is highly induced during infection of *P. sylvestris*, and induces cell death, phytoalexin production, and upregulation of multiple defense genes in the non-host plant *Nicotiana tabacum* (Chen et al., 2015). Recombinant HaCPL2 also induces cell death and inhibits root growth in *P. sylvestris*, accompanied by an upregulation of genes related to jasmonic acid (JA)/ethylene (ET) signaling pathways in *P. sylvestris* (Chen et al., 2015). However, whether HaCPL2 triggers programmed or necrotic-like cell death in its host requires further investigation.

Lipase domain containing proteins constitute another protein family with a role in cell death induction. In AG1IA anastomosis group of *Rhizoctonia solani*, the causal agent of rice sheath blight, the effector AGLIP1 contains this lipase domain and elicits cell death, with or without a functional lipase domain in non-host *N. benthamiana*, but not *Arabidopsis* (Li et al., 2019). Ectopic expression of AGLIP1 in transgenic *Arabidopsis* led to a suppression of defense-response genes, suggesting AGLIP1 also suppresses immune signaling (Li et al., 2019). Whether cell death elicited by AGLIP1 in plants is due to recognition of AGLIP1 by certain plant receptors, direct phytotoxicity, or lipase activity remains to be investigated.

There is a special group of “necrosis-inducing” proteins (NIP) referred to as catalytic NIPs due to their glycosyl hydrolase activities. Another group of NIPs which lack a catalytic domain will be discussed in the next section. Catalytic NIPs not only degrade plant cell walls like CWDEs, but also induce plant defense responses that are often unrelated to their catalytic activity (Kars et al., 2005). The latter property contrasts with endopolygalacturonases, like BcPG1 and BcPG2 discussed above, whose necrotizing activity is dependent on enzymatic activity. Several catalytic NIPs have been reported in *B. cinerea* (Noda et al., 2010; Zhang et al., 2015; Frías et al., 2016; González et al., 2017; Zhu et al., 2017; Yang Y. et al., 2018; Bi et al., 2020). One of these catalytic NIPs, BcGs1, is a glucan 1,4-alpha-glucosidase, and infiltration of BcGs1 in tobacco and tomato leaves induces strong cell death, accumulation of ROS, and upregulation of PR genes, suggesting an HR-like PCD (Zhang et al., 2015). Interestingly, BcGs1-treated plants induce disease resistance against tobacco mosaic virus, *Pseudomonas syringae* pv. tomato DC3000, and against *B. cinerea* itself (Zhang et al., 2015). This study demonstrates that BcGs1 acts as an elicitor that induces a strong, localized immune response that likely primes plant defenses for subsequent infections. However, *B. cinerea* actively secretes BcGs1, which may trigger HR-like PCD at the point of infection, allowing *B. cinerea* to take advantage of the subsequent cell death leading to susceptibility.

*Botrytis cinerea* xylanase BcXyn11A (Noda et al., 2010; Frías et al., 2019) and BcXyl1 (Yang Y. et al., 2018) are catalytic NIPs with xylan degrading activity, but also induce HR-like cell death including ROS production and electrolyte leakage, which is independent of their xylan degrading activity. In addition, a 26-amino acid peptide derived from BcXy1, and a 25-amino acid peptide derived from BcXyn11a are sufficient for eliciting HR-like cell death (Yang Y. et al., 2018; Frías et al., 2019). Recognition of BcXy1 is mediated by plant apoplastic LRR receptor-like kinases BAK1 and SOBIR1 (Yang Y. et al., 2018). Similarly, a xyloglucanase BcXYG1 was reported to have cell death- and immune response-eliciting activities which were also independent of xyloglucanase activity (Zhu et al., 2017).

BcIEB1 from *B. cinerea* is another elicitor that induces HR-related symptoms of cell death, ROS burst, autofluorescence, and electrolyte leakage (Frías et al., 2016). Additional work on BcIEB1 found that this protein, specifically a 35-amino acid conserved region called ieb35, interacts with a plant osmotin, which belongs to family 5 of PR proteins in plant cells and the interaction protected *B. cinerea* from the antifungal osmotin (González et al., 2017). Moreover, osmotin was shown to interfere with the cell death induced by BcIEB1, but was not directly involved in the elicitation of defense responses (González et al., 2017).

### Effectors With Unknown Protein Domains

Recent bioinformatic studies have revealed repertoires of small secreted proteins (SSPs) in several phytopathogens that might contribute to virulence, and many of them are cysteine-rich, small-sized proteins without known protein domains (Wen et al., 2019; Denton-Giles et al., 2020). Predicted effectors with no known domains from a transcriptome study of *S. sclerotiorum* were recently screened for cell death-inducing activity (Seifbarghi et al., 2020). One such effectors, SsNE2, induces cell death in *N. benthamiana* (Seifbarghi et al., 2020). Interestingly, this cell death activity requires plant receptor-like kinases, suggesting a hijacking of immune signaling (Seifbarghi et al., 2020). Similarly, two NIPs without catalytic domains, ZtNIP1 and ZtNIP2, were also identified as putative effectors from *Zymoseptoria tritici* (Ben M’Barek et al., 2015). Further characterization of ZtNIP1 and ZtNIP2 indicated that infiltration of both proteins induces cell death, but only in select wheat cultivars. The genetic basis for susceptibility to these effectors is yet to be determined (Ben M’Barek et al., 2015).

In the conifer pathogen *Heterobasidion parviporum*, a SSP without any known protein domains, HpSSP35.8, was identified as a necrotrophic effector protein secreted during infection (Wen et al., 2019). Overexpression of HpSSP35.8 induces a strong cell death phenotype and activates PR genes, the “WRKY” transcription factors, genes involved in JA/ET-signaling, and chitinase genes, all of which are known defense responses (Wen et al., 2019). This response suggests that the cell death observed is an HR-like response, though HpSSP35.8 also had significant effects on chlorophyll and photosynthesis, which could indicate that the cell death observed is truly necrosis. It is interesting to note that one of the HpSSP3.5-triggered plant responses, the induction of chitinase genes, was also observed in *P. nodorum* SnTox1 - wheat Snn1 interaction, where SnTox1 has chitin binding activity for protecting *P. nodorum* from degradation of induced plant chitinases (Liu et al., 2016). Whether HpSSP35.8 has the same chitin-binding activity is yet to be investigated, though it has no predicted chitin-binding domain.

A comparative analysis of secretomes of three closely related members of Sclerotiniaceae, *B. cinerea*, *S. sclerotiorum*, and *Ciborinia camelliae*, revealed an expansion of a class of cysteine-rich SSPs in the genome of *C. camelliae* (73 CcSSPs) compared to only one homolog in both *B. cinerea* (BcSSP2) and *S. sclerotiorum* (SsSSP3). These are called *C. camelia*-like SSPs (CCL-SSPs) due to their abundance in *C. camelliae* (Denton-Giles et al., 2020). This SSP family is conserved in many other plant pathogens, but for the most part, have not been characterized. Interestingly, recombinant BcSSP2 and SsSSP3 induce significantly stronger and faster cell death than any CcSSP tested (Denton-Giles et al., 2020). Furthermore, both BcSSP2 and SsSSP3 are capable of inducing cell death in plant hosts (camelliae) and non-hosts (*N. benthamiana*) (Denton-Giles et al., 2020), suggesting that CCL-SSPs might act as non-host-specific, broad cell death inducers in a wide range of plants.

### Effectors That Play Roles in Biotrophy-Necrotrophy Switch (BNS)

Because hemibiotrophic fungal pathogens display both biotrophic and necrotrophic qualities, stage specific effectors play a key role in the establishment of infection. Some effectors secreted from hemibiotrophs induce cell death at the later stages of infection, but others facilitate the biotrophy-necrotrophy switch (BNS). During infection of lentil (*Lens culinaris*) by the hemibiotroph *Colletotrichum truncatum*, effector CtNUDIX (NUcleoside DIphosphate linked to some other moiety X) regulates the BNS (Bhadauria et al., 2013). In bacteria and mammals, proteins containing Nudix hydrolase domains catalyze hydrolysis of mutagenic nucleotides and toxic components and function as cellular surveillance enzymes to maintain physical homeostasis (Safrany et al., 1999; Xu et al., 2001; McLennan, 2006). During infection, CtNUDIX is exclusively expressed during the late biotrophic stage precisely at the BNS. CtNUDIX localizes to the plant plasma membrane and induces severe HR-like cell death when overexpressed in *N. benthamiana* (Bhadauria et al., 2013). CtNUDIX-overexpressing strains of *C. truncatum* and *M. oryzae* fail to induce disease symptoms on lentil and barley, respectively, but light-brown discoloration is observed, which is suspected to be HR-like cell death on infected plants (Bhadauria et al., 2013). These results suggest that the timely secretion of CtNUDIX is key to normal disease progression, and its premature secretion likely triggered cell death in the biotrophic phase, preventing these two hemibiotrophs from establishing infection.

Another example of an effector involved in BNS is found in *Colletotrichum graminicola*, the causal agent of anthracnose of maize (Vargas et al., 2012). The *C. graminicola* effector, Cgfl, is a metalloprotease fungalysin that is highly induced during the switch to the necrotrophic stage (Vargas et al., 2012). Sanz-Martín et al. (2016) further characterized this metalloprotease as a predicted chitinase degrading enzyme, and deletion mutants of Cgfl displayed severely reduced virulence on plants. This suggests that Cgfl may be involved in suppressing chitin-triggered immunity as a virulence mechanism, particularly during the BNS when it is most highly expressed.

In *Collectotrichum lentis*, the effector ClToxB is also induced during the BNS in virulent races of *C. lentis* (Bhadauria et al., 2015). Intriguingly, ClToxB shares extensive sequence similarity with PtrToxB from *P. tritici-repentis*, suggesting ClToxB is a potential HST on lentils. Indeed, RNAi strains of *C. lentis* with reduced expression of ClToxB show impaired virulence compared to the wild-type strain, and its transient overexpression failed to induce cell death in the non-host tobacco *N. tobacum* (Bhadauria et al., 2015). Interestingly, if susceptibility to ClToxB is conditioned by an R-like protein, as is the case in the *P. tritici-repentis* pathosystem, this would constitute a remarkable example where R-mediated processes can participate in both resistant and susceptible outcomes in this hemibiotroph depending on the phase of infection. However, further work is required to identify the plant target(s) of ClToxB, and to better understand the biochemical context in which it functions.

### Non-traditional Effectors and Host-Selective Toxins (HSTs)

Fungal secondary metabolism has seen a resurgence of research attention in recent years (Keller, 2019). Many well-studied HSTs are fungal secondary metabolites (SM), and have strong disease-inducing effects in plants (Stergiopoulos et al., 2013; Tsuge et al., 2013). These fungal SM range from small modified peptides to molecules with complex chemical structures, and have various modes of action specific to susceptible host genotypes.

For example, HC-toxin is a cyclic tetrapeptide from *Cochliobolus carborum*, the causal agent of Northern corn leaf spot, that suppresses expression of host defense genes by targeting histone-deacetylases in susceptible plants (Brosch et al., 1995; Walton, 2006). The chlorinated peptides Peritoxin A and B produced by the sorghum pathogen *Periconia circinata* induce apoptotic cell death (Dunkle and Macko, 1995) in sorghum plants harboring NBS-LRR type of R gene *Pc-B* (Nagy and Bennetzen, 2008). This suggests that *P. circinata* deploys peritoxins to exploit PCD mechanisms in sorghum similar to that of victorin in oats (Lorang et al., 2012), though a direct interaction between peritoxins and R-genes has yet to be determined.

Mycotoxins like aflatoxins, trichothecenes, and structurally related toxins are major concerns in food contamination because of their high toxicity to mammals (Arunachalam and Doohan, 2013; Fasoyin et al., 2019; Luciano-Rosario et al., 2020). However, many of these fungal SM also contribute to plant colonization and pathogenicity. For instance, *Aspergillus flavus* and *P. expansum* are common post-harvest pathogens of a broad range of storage crops, and also strong producers of toxic SM (Kelley et al., 2012; Luciano-Rosario et al., 2020). Patulin and citrinin produced by *P. expansum* contribute to aggressiveness and establishment in some plant cultivars, although neither is required for infection (Snini et al., 2016; Touhami et al., 2018; Luciano-Rosario et al., 2020). These post-harvest pathogens rely heavily on secreted CWDE (Fountain et al., 2014), though other proteinaceous effectors and virulence factors have also been identified and are covered in section “Cell Death Inducing Effectors Containing Conserved Domains” (Levin et al., 2019; Luciano-Rosario et al., 2020).

Additional examples of SM that contribute to virulence are found in *Alternaria* and *Fusarium* species. *Alternaria alternata* f. sp. *lycopersici* deploys AAL toxins that induce apoptotic-like phenotypes in sensitive tomato plants (Wang et al., 1996), but not in plants harboring the Asc1 (*Alternaria* stem canker resistance gene 1) gene (Brandwagt et al., 2000). Additional *A. alternata* pathotypes produce similar toxins that have a wide range of negative effects on plant cells, and have been summarized elsewhere (Meena and Samal, 2019). Deoxynivalenol, a mycotoxin produced by *Fusarium graminearum*, accumulates to high concentrations in the necrotic phase of infection and elicits strong ROS burst (Mudge et al., 2006; Desmond et al., 2008), but can actually inhibit PCD at low concentrations (Diamond et al., 2013).

## Plant Immunity-Suppressing Effectors

Accumulating evidence suggests that necrotrophs interact with their hosts in a more subtle and complex way, beyond cell death induction. Recent studies show that some necrotrophic effectors do in fact suppress plant immunity (Table 3), a commonly accepted feature of effectors from biotrophic and early-stage hemibiotrophic pathogens.

**TABLE 3:** Plant immunity-suppressing effectors in necrotrophic fungi.

Pathogen name	Effector	Class/Domain	Function	References
*Cochliobolus carborum*	HC-toxin	Secondary metabolite	Disrupt plant histone deacetylaces	Brosch et al., 1995; Walton, 2006
*Colletotrichum graminicola*	Cgfl	Metalloprotease	Degrade plant chitinases	Vargas et al., 2012; Sanz-Martín et al., 2016
*Sclerotinia sclerotiorum*	SsITL	Integrin-like domain protein	Disruption of calcium signaling, induce SA/suppress JA signaling	Nomura et al., 2012; Tang et al., 2020
	Oxalic acid	Elicitor	Suppress ROS burst and callose deposition	Williams et al., 2011
	SsEWCA	Chitinase	Suppress chitin-triggered immunity	Martínez-Cruz et al., 2021
*Botrytis cinerea*	Bc-siR3.1	Small RNA	Reduce expression of key immune signaling genes	Weiberg et al., 2013
	Bc-siR3.2	Small RNA	Reduce expression of key immune signaling genes	Weiberg et al., 2013
	Bc-siR5	Small RNA	Reduce expression of key immune signaling genes	Weiberg et al., 2013
	Bc-siR37	Small RNA	Reduce expression of key immune signaling genes	Wang et al., 2017
	BcEWCA	Chitinase	Suppress chitin-triggered immunity	Martínez-Cruz et al., 2021
*Rhizoctonia solani*	RsLysM	LysM containing protein	Chitin binding	Wibberg et al., 2016; Dölfors et al., 2019
	RsRlpA	Lipoprotein	Protease inhibition	Charova et al., 2020
	RsEWCA	Chitinase	Suppress chitin-triggered immunity	Martínez-Cruz et al., 2021
*Valsa mali*	VmEP	Unknown	Unknown	Li et al., 2015
*Verticillium dahilae*	VdPDS1	Chitin deacetylase	Modify chitin to non-immunogenic chitosan	Gao et al., 2019

### Effectors Preventing Pathogen Recognition and Immunity

The lysin motif (LysM) domain is a widespread protein motif found in many organisms, including fungi and plants, and is known for binding peptidoglycans and chitin (Kaku et al., 2006; Miya et al., 2007; Buist et al., 2008; Shimizu et al., 2010). During infection, secreted plant chitinases lead to degradation of fungal cell walls, releasing fungal cell wall fragments like chitin or chitosan oligomers. These oligomers are detected by plant extracellular receptors with LysM domains, thus triggering an immune response (Thomma et al., 2011). Many biotrophic and hemibiotrophic fungal effectors also contain the LysM motif and competitively bind to chitin or its oligomers, interfering with plant recognition of these oligomers (de Jonge et al., 2010; Marshall et al., 2011; Kombrink et al., 2017). A recent study in the necrotrophic fungus *R. solani* found that RsLysM, the only putative LysM effector present in the genome of *R. solani* AG2-2IIIB (Wibberg et al., 2016), suppresses chitin-triggered immunity by binding to chitin (Dölfors et al., 2019). Notably, RsLysM does not protect hyphae from chitinolytic activity of plant chitinases (Dölfors et al., 2019), as some other chitin-binding effectors like *Cladosporium fulvum* Avr4 does, but likely prevents recognition by plant chitin receptors (van den Burg et al., 2006). A new family of chitin-related effectors were recently identified in the biotrophic fungus *Podosphaera xanthii* which causes powdery mildew in cucurbits, and termed effectors with chitinase activity (EWCA) (Martínez-Cruz et al., 2021). These EWCA degrade chitin oligomers, therefore suppressing chitin-triggered immunity. Phylogenetic analyses revealed that EWCA orthologs are present in many necrotrophic fungi too, including *B. cinerea*, *S. sclerotiorum*, and *R. solani* (Martínez-Cruz et al., 2021). Whether these EWCA orthologs also act as inhibitors of chitin-triggered immunity in necrotrophs is yet to be determined. Similarly, a polysaccharide deacetylase (PDA1) effector was recently identified in the hemi-biotrophs *Verticillium dahliae* and *F. oxysporum*, which converts chitin to chitosan thus preventing chitin-triggered immune signaling in cotton (Gao et al., 2019). VdPDA1 is highly expressed early in infection and likely contributes to a successful biotrophic phase of *V. dahlia* infection, as PDA1 knockout mutants had severely reduced virulence (Gao et al., 2019).

Another *R. solani* AG2-2IIIB effector protein, RsRlpA, encodes a rare lipoprotein A-like that has immunity suppressing activity (Charova et al., 2020). RsRlpA is highly induced during early infection of sugar beets, and its overexpression in *N. benthamiana* suppresses HR imposed by *C. fulvum* Avr4 (Charova et al., 2020). RsRlpA shares sequence homology to papain-like inhibitors which are known for blocking activity of papain-like cysteine proteases (PL) that help induce PCD and other immune responses in plants (reviewed by Misas-Villamil et al., 2016). Indeed, the HR inhibition by RsRlpA is associated with protease inhibitor activity, inhibiting a plant cathepsin that is a known PLCP (Bárány et al., 2018). Various other plant protease activities, including caspase-like activities, have been shown to function in the execution of plant PCD (Kabbage et al., 2017), so suppression of HR-like cell death through other protease inhibition mechanisms may also be present in plant-pathogen interactions. AGLIP1 from *R. solani* strain AG1IA, which was mentioned in the previous section, also inhibits plant immunity, but by currently unknown mechanisms (Li et al., 2019).

In the apple Valsa canker fungus, *Valsa mali*, seven out of 70 randomly selected candidate effectors (referred to as VmEPs) were shown to suppress BAX-induced PCD in *N. benthamiana* (Li et al., 2015). The Bax protein belongs to the cell death antagonist Bcl-2 family of proteins and was demonstrated to induce cell death resembling defense-related HR in plants (Lacomme and Santa Cruz, 1999; Kabbage et al., 2017). Suppression of Bax-associated PCD by VmEPs indicates that these effectors inhibit plant HR-related resistance during infection.

Overall, the suppression of cell death in these examples are counterintuitive considering the trophic lifestyle of these pathogens. However, we speculate that early stages of infection by necrotrophs may require a suppression of cell death to establish infection before triggering the opposite, in a manner that is analogous to hemibiotrophic infections. Indeed, the prototypical necrotroph *S. sclerotiorum* is proposed to have a brief biotrophic phase before quickly switching to necrotrophy (Kabbage et al., 2015). Alternatively, it may be possible that not all cell deaths are created equal, and the type of cell death triggered by the plant may differ from the one imposed by the pathogen, and therefore needs to be suppressed. In accordance, an interplay between autophagic and apoptotic cell deaths with opposing outcomes was reported in necrotrophic fungal infections (Kabbage et al., 2013), and a deeper understanding of the native plant PCD mechanisms is needed (Kabbage et al., 2017).

### Effectors Altering Hormone, Calcium Signaling, and Oxidative Burst for Improper Immune Responses

Phytohormones play critical roles in disease resistance and in general, salicylic acid (SA) signaling pathways are induced for successful resistance against biotrophs (Glazebrook, 2005), while induction of JA signaling pathways typically lead to successful defense against necrotrophs (Gfeller et al., 2010). As extensive crosstalk between the SA and JA signaling pathways have been demonstrated (Kunkel and Brooks, 2002), it’s reasonable to expect pathogen effectors to manipulate hormone signaling pathways to suppress hormone mediated resistance (El Oirdi et al., 2011). For example, fungal integrin-like (ITL) proteins are important for fungal development (Corrêa et al., 1996; Zhu et al., 2013), but SsITL from *S. sclerotiorum* is secreted during plant infection and interacts with chloroplast-localized calcium sensing receptor, promoting SA biosynthesis (Nomura et al., 2008, 2012; Tang et al., 2020). This influx of SA leads to a suppression of JA signaling, culminating in enhanced susceptibility to *S. sclerotiorum*. In a hypovirulent strain of *S. sclerotiorum*, SsITL is significantly downregulated, supporting its role in virulence (Li et al., 2008).

A non-traditional effector produced by *S. sclerotiorum* that leads to improper immune responses is oxalic acid (OA). The effects of OA are broad, initially creating a strong reducing environment that dampens immune responses like ROS burst, callose deposition, and autophagy (Williams et al., 2011; Kabbage et al., 2013). Later in infection, accumulation of OA can induce ROS and trigger apoptotic-like PCD, contributing to necrotrophic success (Kabbage et al., 2013).

## sRNA Effector-Like Molecules

Recent studies have revealed that non-coding small RNAs (sRNAs) derived from necrotrophic fungi can also be delivered into host cells, hijack plant RNAi machinery, and silence plant genes that are involved in immunity (Weiberg et al., 2013; Wang et al., 2016, 2017). As small, secreted molecules that affect plant physiology, sRNAs are now often considered effector-like molecules, despite being non-proteinaceous (Weiberg and Jin, 2015). Pathogen sRNAs are mainly derived from gene-poor, repeat-rich regions in genomes (Weiberg et al., 2013) and plant targets of sRNAs are typically associated with immune responses (Dong et al., 2015).

To date, sRNA effectors from necrotrophic fungi have largely been reported from *B. cinerea*. The sRNAs Bc-siR3.1, Bc-siR3.2, and Bc-siR5 are the most abundant sRNAs during infection of tomato and *Arabidopsis* (Weiberg et al., 2013). These sRNAs specifically target a peroxiredoxin (oxidative stress-related gene), mitogen activated protein kinases (MPK1, MPK2, MPKKK4), and a cell wall-associated kinase (WAK) (Weiberg et al., 2013). These plant targets of fungal sRNAs are known components of immune responses; oxidative burst and signal transduction pathways. Thus, their silencing results in enhanced susceptibility to the fungus (Weiberg et al., 2013). Another sRNA, Bc-siR37, can lead to silencing of *Arabidopsis* WRKY transcription factors, receptor-like kinases, and cell wall modifying enzymes, all leading to suppression of plant immunity against the fungus (Wang et al., 2017).

In *S. sclerotiorum*, sRNA sequencing of the fungus *in vitro* and during infection of *Arabidopsis* and common bean (*Phaseolus vulgaris*) revealed a group of fungal sRNAs that were secreted specifically *in planta* (Derbyshire et al., 2019). These sRNAs are predicted to target and suppress plant genes that are associated with quantitative disease resistance during infection (Derbyshire et al., 2019). In particular, mutations of two sRNA targets, encoding kinase genes SERK2 and SNAK2, increased susceptibility to *S. sclerotiorum*, suggesting that these sRNA targets contribute to disease resistance (Derbyshire et al., 2019). The role of sRNA in plant-pathogen interactions is an emerging field of plant pathology, and continued advances in sequencing technologies followed by functional characterization will likely reveal a broad utilization of these effector-like molecules across fungal taxa.

## Conclusion and Future Perspectives

**FIGURE 1:**
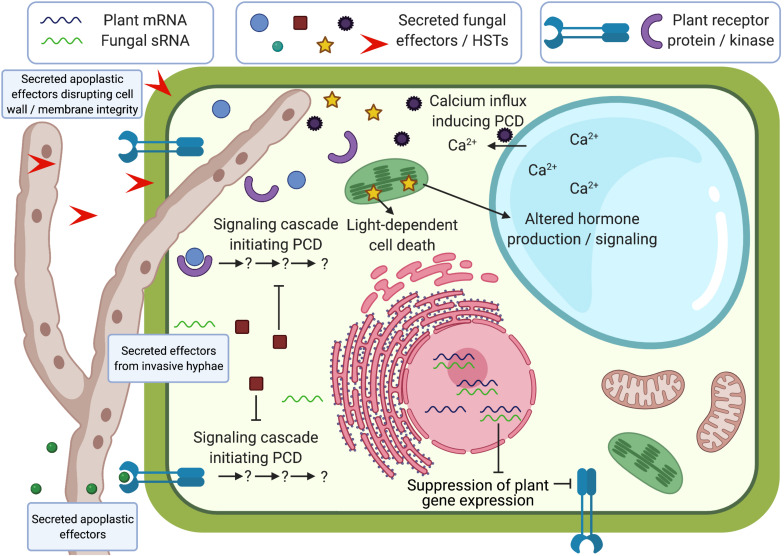
Schematic representation of effector functions during plant-fungal necrotroph interactions. Effectors can be secreted into the apoplast or into plant cells from invasive hyphae, and can disrupt cell wall/membrane integrity, initiate programmed cell death (PCD), suppress PCD, alter hormone signaling, affect signaling cascades that use calcium ion fluxes, or suppress plant gene expression through small non-coding RNAs (sRNAs). Some of these processes have been shown to be light-dependent. Created with BioRender.com.

Necrotrophic fungal pathogens of plants were previously considered to cause cell death with simplistic mechanisms by secreting phytotoxic molecules and degrading plant cell walls. Recent bioinformatic advances and functional studies have accelerated the discovery of virulence factors in necrotrophs, including proteinaceous effectors, HSTs, and sRNA effectors, revealing that necrotrophs utilize a broad range of sophisticated virulence mechanisms during infection of plants (Figure 1). Our understanding of necrotrophic effectors is improving and studies have revealed that these molecules are capable of both subverting and hijacking plant physiological processes to their advantage, including PCD. While PCD mechanisms are successfully deployed by plants that recognize biotrophic and hemibiotrophic effectors, necrotrophic effectors evolved to manipulate plant PCD and other immune responses to promote susceptibility. As necrotrophic fungi continue to cause significant crop losses worldwide, it is essential to improve our understanding of these molecules and their plant targets to identify novel modes of resistance against these pathogens.

To date, qualitative and robust genetic resistance to necrotrophic fungi is lacking in plants, aside from a few plant genotypes that are insensitive to fungal HSTs. Similarly, quantitative resistance against necrotrophs has shown limited efficiency and complicate breeding efforts. However, the recent discovery of sRNA cross-talk between plants and fungi has opened new avenues for disease control. Multiple fungal pathogens have now been shown to uptake environmental RNAs, leading to the silencing of specific fungal genes. Thus, plants can be weaponized to target virulence factors of necrotrophic fungi, including necrotrophic effectors. Spray-induced gene silencing is also showing promise as an RNAi tool.

Lastly, while many necrotrophic effectors manipulate plant PCD, the biochemical context of PCD in plants is poorly understood (Kabbage et al., 2017). We propose that necrotrophic effectors can be used as a valuable tool to uncover plant PCD components. A mechanistic understanding of how effectors trigger PCD, and how PCD can be prevented, is likely to have implications beyond plant-fungal interactions. In conclusion, there is a wealth of potential applications of plant-fungal necrotrophic effector research in disease control, basic plant physiology, and fungal biology, so expanding our understanding of these molecules will greatly expedite these applications.

## Author Contributions

DS and MR reviewed and evaluated the relevant literature, generated the figures, and created the tables. DS wrote the manuscript with significant contributions from MR, MK, and DLS. All authors contributed to the article and approved the submitted version.

## Conflict of Interest

The authors declare that the research was conducted in the absence of any commercial or financial relationships that could be construed as a potential conflict of interest.

## Abbreviations

PCD: programmed cell death
CWDE: cell wall degrading enzymes
HR: hypersensitive response
HST: host selective toxin
QTL: quantitative trait loci
OA: oxalic acid
PR: pathogenesis related
ROS: reactive oxygen species
BNS: biotrophy/necrotrophy switch
SM: secondary metabolites
sRNAs: non-coding small RNAs.
